# In-air fluence profiles and water depth dose for uncollimated electron beams

**DOI:** 10.4103/0971-6203.44473

**Published:** 2008

**Authors:** Abedelkadar Toutaoui, Amar Nassim Aichouche, Kenza Adjidir Adjidir, Ahmed Chafik Chami

**Affiliations:** Départment de Physique Médicale, Centre de Recherche Nucléaire d' Alger, Algiers, Algeria; 1 Laboratoire de Sciences Nucléaires, Faculté de Physique, Université des Sciences et de la Technologie Houari Boumedienne, BP 32 El Alia, Bab Ezzouar, Algiers, Algeria; 2 Bd Frantz Fanon BP399 Alger RP, Algiers, Algeria

**Keywords:** Monte Carlo electron beam algorithm, phase space model, uncollimated electron beams

## Abstract

Advanced electron beam dose calculation models for radiation treatment planning systems require the input of a phase space beam model to configure a clinical electron beam in a computer. This beam model is a distribution in position, energy, and direction of electrons and photons in a plane in front of the patient. The phase space beam model can be determined by Monte Carlo simulation of the treatment head or from a limited set of measurements. In the latter case, parameters of the electron phase space beam model are obtained by fitting measured to calculated dosimetric data. In the present work, data for air fluence profiles and water depth doses have been presented for electron beams without an applicator for a medical linear accelerator. These data are used to parameterize the electron phase space beam model to a Monte Carlo dose calculation module available in the first commercial (MDS Nordion, now Nucletron) Monte Carlo treatment planning for electron beams.

## Introduction

Advanced 3D electron beam dose calculation models such as the phase-space evolution model[1–3] and several `macro´ Monte Carlo[4–6] simulations require the input of an initial phase space (IPS) that describes a clinical electron beam. Such initial phase spaces describe the electron beam, the differential in space co-ordinates perpendicular to the beam axis, and the differential in the energy and angle.

An initial phase space is calculated using the simulation of electron transport through the head of a clinical linear accelerator and registers the electrons that enter the IPS plane. The EGS4/BEAM Monte Carlo code[7] is an implementation of this method. The results of this simulation can be parameterized by a multiple-source model[8] having the advantage of substantially less storage capacity.

As an alternative to the above method, simple models for the initial phase space have been proposed that are fully based on measurements.[9] Methods based on only a limited set of measured beam data have an advantage in clinical practice as they can be easily implemented in treatment planning systems.

In 2001, the first commercial Monte Carlo treatment planning for electron beams was released by MDS Nordion (now Nucletron), which has been implemented in the Dose Calculation Module (DCM) and can be accessed from the THERAPLAN PLUS™ or ONCENTRA treatment planning systems.

The implementation of the Monte Carlo calculation algorithm for electrons has two independent components: (i) a new, coupled Multi-Source electron beam model[10–12] for electron transport through the linear accelerator treatment head, and (ii) a Monte Carlo electron and photon transport dose calculation code for inpatient dose calculation, the VMC++ Monte Carlo algorithm developed by Kawrakow and co-workers.[5,13–15]

The coupled multisource beam model dealing with the electron transport through the accelerator head is based on an energy distribution of the electrons specified by a laterally invariant energy spectrum and small-angle five-parameter parameterization. This describes the effective lateral and directional distributions of electrons.

The energy spectrum is determined by minimizing the difference between measured and calculated water depth doses from a large field depth dose measurement in an open electron beam (without an applicator). The parameters of the electron phase space beam model are obtained by fitting measured to calculated open field air fluence profiles for a set of rectangular field shapes defined by the block collimators.

In this work, measurement of air fluence profiles and water depth doses have been presented for electron beams without the applicator. These data were collected, for the radiation beam characterization of the THERAPLAN PLUS™ treatment planning system installed in the Radiotherapy Department of the Centre Pierre and Marie Curie, Algiers. The set of beam characterization dosimetric data includes, amongst other things, measured profiles and depth dose in air and water for each beam energy, both without and with the applicators on. The beams have been “fitted” by the vendor and the TPS is provided with the ready-to-use virtual treatment units.

## Materials and Methods

### Incident beams and detectors

Measurements were performed in a Clinac 1800 linear accelerator (Varian) which produces nominal electron energies from 6 to 20 MeV. A special nonclinical mode (e.g., service mode) is used to perform the measurements for electron beams without an applicator, over-riding interlocks of the machine. This was necessary for the measurements described in this paper, as well as for commissioning electron beams.[1,5]

A commercially available radiation field analyzer (Scanditronix-Wellhofer RFA200, Uppsala, Sweden) was used to obtain all measurements in air and water. The relative dose was measured using a p-type silicon diode. A monitoring detector (similar diode) was used as a reference detector in air near the edge of the field to account for any effects of beam variations. Measurements were made in the continuous scanning mode with 1 mm resolution. The estimated overall relative uncertainties in our measurements were within 2%.

### Air fluence measurements

Air fluence measurements were carried out in free geometry air without any electron applicator on the accelerator.

In-air measurements were performed using an electron diode without any build-up cap. The detector was located in a scanning device at the maximal height possible in an empty water phantom. Measurements were carried out along the central axis and in horizontal planes at two different source-to-detector distances (SDD). For each electron beam energy, measurements were done for rectangular fields which consist of air fluence profiles along the in-plane and cross-plane directions for both source-to-detector distances (SDD), as well as a depth dose measurement along the z-axis starting above the upper measurement plane (corresponding to the first SDD) and extending beyond the lower plane (corresponding to the second SDD).

For the rectangular fields, the setting of the uppermost jaw was fixed, defining an 8 cm slit field projected at isocenter plane. The lower jaw was varied to define widths of 8, 20, and 35 cm fields.

In addition to the above, measurements were also performed for a large square field of 35 × 35 cm^2^ area to measure the full penumbra.

For each field, measurements were carried out in two planes located 85 and 105 cm from the nominal target position, and these measurements covered the 90–10% penumbra plus at least 4 cm. In addition to this, a scan along the central axis was acquired that was parallel with the radiation beam This scan starts 2 cm above the first SDD and extends 5 cm beyond the second SDD.

The spatial increment for the profile measurements in high gradient regions such as the field edges the scan step not exceed 0.1 cm and in low gradient regions, this increment was increased to 0.2 cm. The measurements along the central axis were performed with a spatial increment not exceeding 0.1 cm.

It was recommended that all in-air profiles have a common normalization point that was not necessarily in absolute units. For the sake of convenience, all in-air profiles were normalized to the signal from the 8 cm × 20 cm field on the central axes for the shorter SDD.

### Dose measurements in water without applicator

The depth dose measurements were performed using a Scanditronix RFA200 dosimetry scanning system and a Scanditronix electron p-type diode detector. The diode signal was normalized, for each energy, at the normalization depth specified above. An open field (no applicator, 35 cm × 35 cm field size) depth dose measurement at SSD = 100 cm was acquired for each energy and without applicator. The measurements were normalized to 100.0 at the normalization depth and extend beyond the depth for the bremsstrahlung measurement listed in Table 1. These depth dose data will be used for deriving the energy spectrum for the phase space model.

**Table 1:** Normalization depths for depth doses and profiles in water and depths for Bemsstrahlung measurements

*Nominal electron energy, Enom (MeV)*	*Normalization depth, dnorm (cm)*	*Depth for Bremsstrahlung measurments, dbre (cm)*
4 MeV ≤ Enom ≤ 6 MeV	1.0	5.0
6 MeV < Enom ≤ 15 MeV	2.0	10.0
15 MeV < Enom ≤ 25 MeV	3.0	15.0
25 MeV < Enom ≤ 35 MeV	3.0	20.0
35 MeV < Enom ≤ 50 MeV	3.0	30.0

## Results

**Figure 1:**
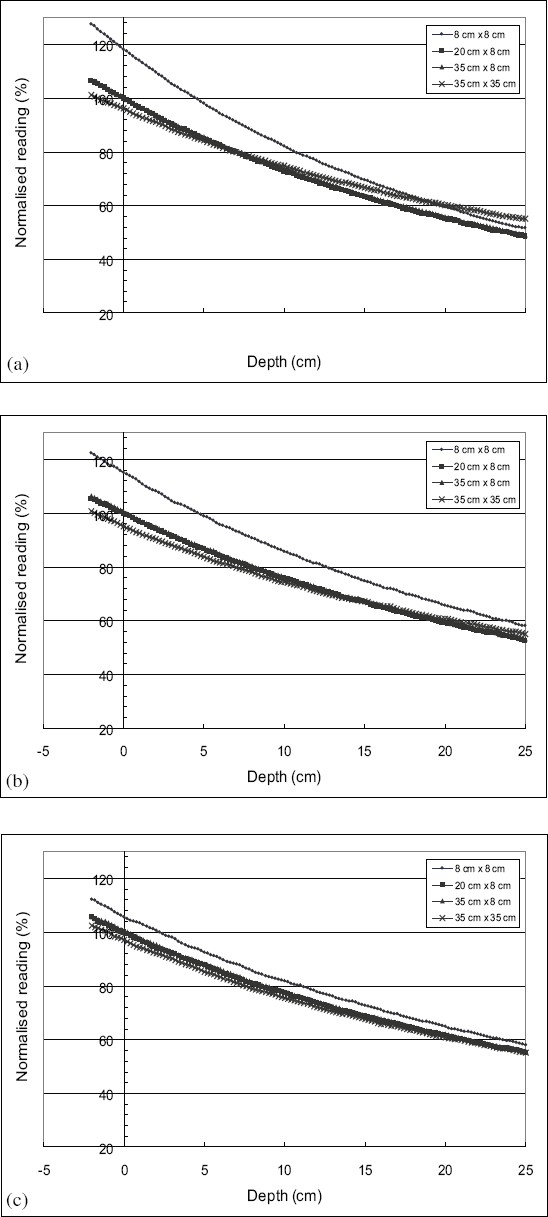
In-air depth dose distributions along the z axis for electron beam energy of (a) 6, (b) 12, and (c) 20 MeV for field sizes of 8 × 8 cm^2^, 20 × 8 cm^2^, 35 × 8 cm^2^, and 35 × 35 cm^2^

The in-air depth dose distributions along the z axis that have been presented in Figure 1a–c for field sizes of 8 × 8 cm^2^, 20 × 8 cm^2^, 35 × 8 cm^2^, and 35 × 35 cm^2^ for electron beam energies 6, 12, and 20 MeV respectively. For all electron beam energies, it is seen that the central axis depth dose for a field size of 8 × 8 cm^2^ presents values greater than those of the depth dose curves of other field sizes.

The difference between the values of the depth dose curve for the 8 × 8 cm^2^ field and those of the depth dose curves for the 20 × 8 cm^2^, 35 × 8 cm^2^, and 35 × 35 cm^2^ (presented in Figure 1a–c) fields at the point nearest to the treatment head (located at 83 cm SSD), is 20% for the electron beams of energy 6 and 9 MeV, of 15% for the beam of 12 MeV, of 10% for the beam of 16 MeV, and 6% for the beam of 20 MeV. These differences are reduced to approximately 5% at the end of the curve for all electron beam energies.

The values of the in-air depth dose curves for the 20 × 8 cm^2^, 35 × 8 cm^2^, and 35 × 35 cm^2^ fields [Figure 1a–c] are close between them. When the electron beam energy increases, these in-air depth dose curves approach each other.

From the results presented in Figure 1, it appears clearly that the slope of the depth dose decreases when the energy of the electron beam increases. With the increase of the beam energy, the scattering of the electrons in the air decreases.

**Figure 2:**
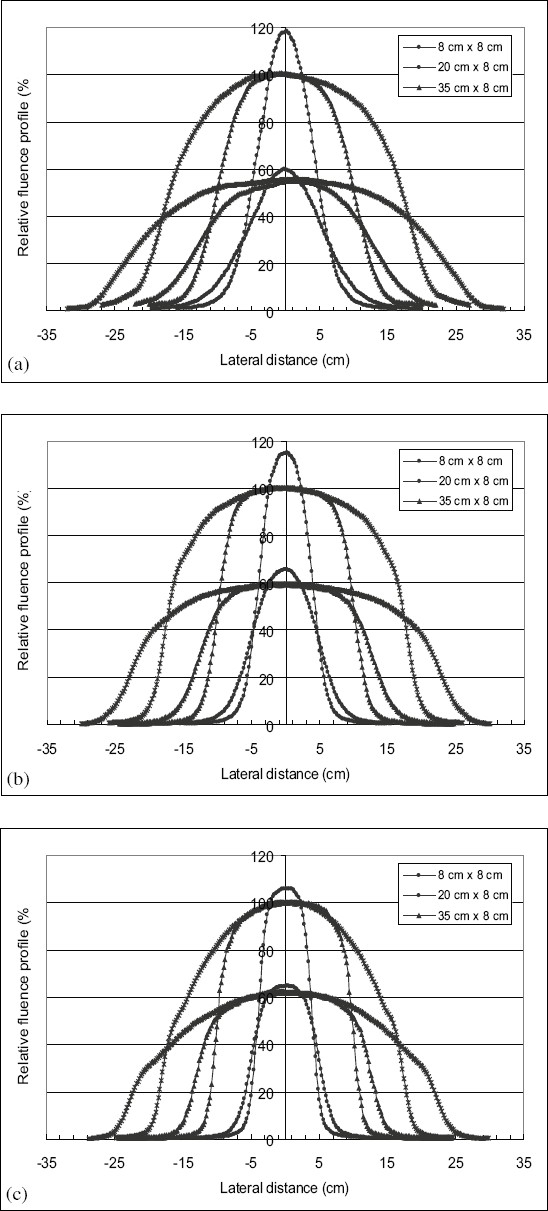
In-plane air fluence profiles measured for rectangular fields at two different distances from the source of the beam (85 and 105 cm) for electron beams energies of (a) 6, (b) 12, and (c) 20 MeV, and field sizes of 8 × 8 cm^2^, 20 × 8 cm^2^, and 35 × 8 cm^2^

Figure 2 shows in-air fluence profiles in the in-plane direction measured for field sizes of 8 × 8 cm^2^, 20 × 8 cm^2^, and 35 × 8 cm^2^ rectangular fields, at two distances, 85 and 105 cm, from the source of the beam, for electron beam energies of 6, 12, and 20 MeV. In Figure 2, it is also seen that the values of the in-air fluence on the central axis for the field size of 8 × 8 cm^2^ are larger than the in-air fluence values for field sizes of 20 × 8 cm^2^ and 35 × 8 cm^2^. Table 2 shows the ratios of the dosimeter readings at the central axis at the distance of measurement of 85 cm, for the different field sizes, normalized to that of 8 × 8 cm^2^ for various electron beam energies. Figure 2 illustrates that the in-air fluence profiles do not exhibit a flat area in the central part of the field for the field size of 8 × 8 cm^2^ and show a narrow flat area for the other field sizes in contrast to what could be observed for the in-air fluence profiles measured for collimated beams.

**Table 2:** Ratios of the readings at the distance of measurement of 85 cm for the different field sizes normalized to that of 8 × 8 cm^2^ for various electron beam energies

*Field size (cm^2^)*	*Normalized readings*
6 MeV	
8 × 8	1.184
20 × 8	1.000
35 × 8	1.003
9 MeV	
8 × 8	1.183
20 × 8	1.000
35 × 8	1.004
12 MeV	
8 × 8	1.152
20 × 8	1.000
35 × 8	1.005
16 MeV	
8 × 8	1.092
20 × 8	1.000
35 × 8	1.007
20 MeV	
8 × 8	1.058
20 × 8	1.000
35 × 8	1.006

**Figure 3:**
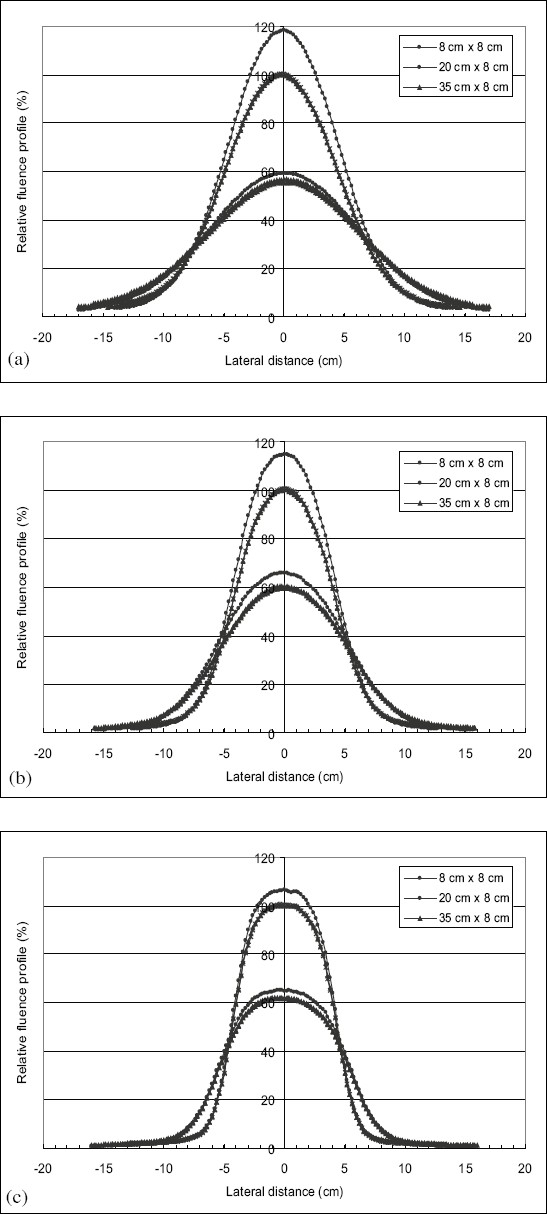
Profiles of in-air fluence measured in the cross-plane direction for the same field sizes and same electron beam energy at the same depths as the measurements of Figure 2

Figure 3 illustrates the profiles of in-air fluence measured in the cross-plane direction for the same field sizes, same electron beam energies, and at the same depths. It can be seen clearly that when the energy of the beam increases, the flat area of in-air fluence profiles widens at the level of the central axis. It is seen that the widening of the profiles of in-air fluence decreases when the energy of the beam increases.

**Figure 4:**
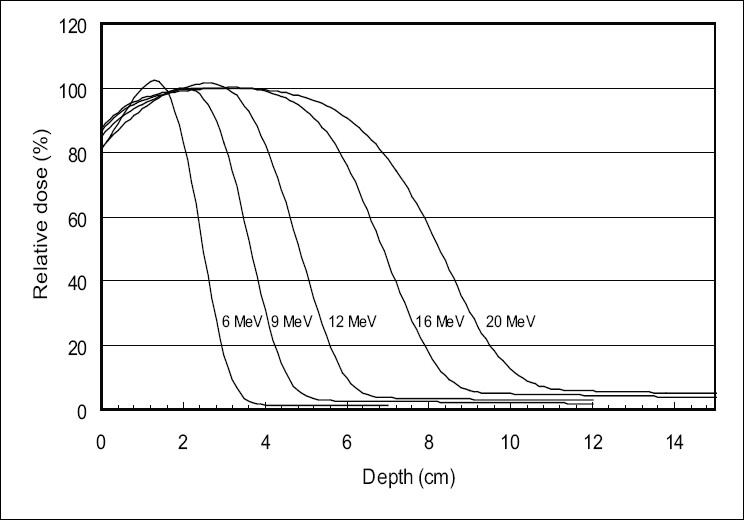
Depth dose measurements for an open field of 35 cm × 35 cm area at 100 cm SSD for 6, 9, 12, 16, and 20 MeV without an applicator

Figure 4 shows the measured depth doses in water for an open field of 35 cm × 35 cm area at 100 cm SSD, for 6, 9, 12, 16, and 20 MeV without an applicator.

The depth dose in water of uncollimated electron beams show a depth of the maximal dose and a practical range larger than those of the collimated beams.

## Discussion

For the in-air depth dose curves, as shown in Figure 1a–c, the central axis depth dose for a field size of 8 × 8 cm^2^ presents values greater than those of the depth dose curves of the other field sizes. This is mainly due to the contribution of the electrons scattered by the jaws of the collimators of photons. When the opening of these jaws increases beyond a certain size, the larger distance the scattered electrons have to traverse does not enable them to reach the central axis. The maximal difference is observed at the level of the point nearest to the treatment head. This variation tends to decrease with the distance of the point of measurement to the electron source for a given electron beam energy.

It was noticed from Figure 2a–c that the in-air fluence profiles do not exhibit or have a narrow flat area in the central part of the field. This is due to the lack of electrons scattered by the walls of the applicator. This justifies the parameterization of the phase space beam model from these profiles of fluence which represent the contribution of direct electrons and to a lesser degree, the electrons scattered by the jaws of the photon collimators.

The widening of the flat area of the in-air fluence profiles at the level of the central axis, with the increase in electron beam energy can be observed in Figure 3a–c. This widening can be explained by the fact that for high energy electron beams, the contribution of the electrons scattered by the jaws of photon collimators becomes more important. Their range becomes sufficient and this enables them to reach the central part of the beam.

The depth dose curves in water measured for the 35 × 35 cm^2^ field size for an electron beam without applicator will be used in the radiation data characterization process for deriving the energy spectra for unscatterd electrons as a part of the electron phase space beam model.

## Summary and Conclusion

This article presents a set of measured in-air fluence and depth dose in water for an uncollimated electron beam, as a part of the dosimetric data set for the commissioning of a Monte Carlo-based treatment planning for electron beams. These data are dedicated to the parametrization of the phase space beam model.

The choice of these data is governed by the requirement of the dose calculation algorithm to model the direct electron and the electrons scattered by the walls of the applicator as well as for the determination of the energy spectrum of the direct beam.

The phase space beam model parameters are derived as part of the treatment unit characterization process by optimizing calculated data towards measured open field data (no electron applicator mounted on the treatment head).

The energy spectrum is determined by minimizing the difference between measured and calculated water depth doses.

These data were sent to the manufacturer for parameterization beams and then built in into the TPS which is in the course of commissioning.
